# Investigating the Role of Individual Differences in Adherence to Cognitive Training

**DOI:** 10.5334/joc.315

**Published:** 2023-08-22

**Authors:** Domenico Tullo, Yi Feng, Anja Pahor, John M. Cote, Aaron R. Seitz, Susanne M. Jaeggi

**Affiliations:** 1 University of California Irvine, Irvine, USA; 2 Univerza v Mariboru, Maribor, Slovenia; 3 Northeastern University, Boston, USA; 4 University of California Riverside, Riverside, USA

**Keywords:** cognitive training, adherence, learning, engagement, individual differences

## Abstract

Consistent with research across several domains, intervention adherence is associated with desired outcomes. Our study investigates adherence, defined by participants’ commitment to, persistence with, and compliance with an intervention’s regimen, as a key mechanism underlying cognitive training effectiveness. We examine this relationship in a large and diverse sample comprising 4,775 adults between the ages of 18 and 93. We test the predictive validity of individual difference factors, such as age, gender, cognitive capability (i.e., fluid reasoning and working memory), grit, ambition, personality, self-perceived cognitive failures, socioeconomic status, exercise, and education on commitment to and persistence with a 20-session cognitive training regimen, as measured by the number of sessions completed. Additionally, we test the relationship between compliance measures: (i) spacing between training sessions, as measured by the average time between training sessions, and (ii) consistency in the training schedule, as measured by the variance in time between training sessions, with performance trajectories on the training task. Our data suggest that none of these factors reliably predict commitment to, persistence with, or compliance with cognitive training. Nevertheless, the lack of evidence from the large and representative sample extends the knowledge from previous research exploring limited, heterogenous samples, characterized by older adult populations. The absence of reliable predictors for commitment, persistence, and compliance in cognitive training suggests that nomothetic factors may affect program adherence. Future research will be well served to examine diverse approaches to increasing motivation in cognitive training to improve program evaluation and reconcile the inconsistency in findings across the field.

## Introduction

The past two decades have witnessed a surge of interest in evaluating the potential of cognitive training to enhance cognition (Katz, Shah, et al., 2018). This interest stems from a rapidly mounting body of evidence indicating that repeated practice on a cognitive task can lead to improvements in non-trained targeted outcomes (Green et al., 2019). However, researchers who question the validity of cognitive training point to the inconsistent findings and small effect sizes that are present across diverse approaches (e.g., Melby-Lervåg & Hulme, 2015; Simons et al., 2016). Furthermore, these inconsistencies are used to challenge the viability and generalizability of cognitive training more broadly (Gobet & Sala, 2023). To better understand the reasons for these inconsistencies and to further advance the understanding of cognitive training effects, the field is moving towards research that identifies the underlying mechanisms that give rise to training-induced gains (Tullo & Jaeggi, 2022). For instance, a special interest within the field of cognitive training is focused on investigating the role of within-subjects factors elucidating the translation of benefits from training gains to targeted outcomes (e.g., Donk et al., 2017; Jaeggi et al., 2014; Karbach et al., 2017; Katz et al., 2016; Shah et al., 2012; Traut et al., 2021). The pursuit of characterizing such factors can result in further tailoring cognitive training approaches to catalyze training benefits and to help reconcile the inconsistency in cognitive training research.

To better understand the mechanisms of cognitive training, one approach is to identify how people engage with intervention materials and protocols to optimize the benefits. For instance, non-adherence to treatment is a common challenge faced by many types of interventions across various domains, such as pharmacological treatments (e.g., Biederman et al., 2020; Chacko et al., 2010; Gau et al., 2008), behavioral therapy (e.g., Farrer et al., 2014; Johansson et al., 2015; Neil et al., 2009), or physical exercise (e.g., Collado-Mateo et al., 2021). Here, we investigate treatment adherence, captured as the degree to which a patient commits to, persists in, and complies with the recommended therapeutic regimen, as specified by the intervention’s established protocols. This mechanism is especially relevant in cognitive training interventions, where adherence to the training regimen may play a crucial role in determining the efficacy of the treatment paradigm (Harrell et al., 2021).

For one, non-commitment and a lack of persistence can have substantial effects on the interpretation of research trials studying the treatment’s efficacy and effectiveness (Jaeggi et al., 2014; Katz, Jaeggi, et al., 2018). The field of cognitive training has employed several strategies to overcome issues of attrition, such as adding a layer of gamification to the cognitive training paradigm (Deveau et al., 2014, 2015; Green & Seitz, 2015; Mohammed et al., 2017; Prins et al., 2010), personalized coaching (Chacko et al., 2010; Nelwan et al., 2018), and positively framing the context of the intervention (Harrell et al., 2021). While most research has focused on developing methods to improve persistence and reduce attrition and dropout, research aimed at identifying individual difference factors that predict and explain commitment to and persistence with treatment, are sparse, inconsistent, and restricted to specific populations.

Our review of the literature reveals a scarcity of studies that investigate the relationship between individual difference factors and persistence in cognitive training interventions. For example, Double and Birney (2016) found that age showed a positive association with treatment perseverance, with older participants demonstrating higher persistence compared to younger participants, while personality traits such as openness were negatively related to treatment perseverance. Additionally, Cruz et al. (2014) reported significant differences in time spent training between individuals with neurodegenerative diseases and brain injuries compared to those without these conditions. Moreover, He et al. (2022) found that memory measures were the most significant predictors of overall persistence. Additionally, a recent study has demonstrated a link between weekly exercise and treatment adherence (Coley et al., 2019). This recent study by Coley et al., (2019) has also suggested that education could predict participants continued participation; nevertheless, the findings were inconsistent between certain levels of education and may have been influenced by the longitudinal design of the study.

In contrast, however, Turunen et al. (2019) found no link between age, sex, or health with dropout and attrition; while Lam et al. (2015) and Cruz et al. (2014) found no link between education and time committed to training. Overall, the extant literature demonstrates inconsistency among individual difference factors predicting commitment and persistence; but most of the aforementioned studies focus on older adults, and thus, it is unclear to what extent their findings might generalize to other populations (Cruz et al., 2014; Double & Birney, 2016; He et al., 2022; Turunen et al., 2019). Furthermore, to our knowledge, no study has clarified the roles of grit, ambition, and socioeconomic status in committing and/or persevering through a cognitive training program. As such, indicators of treatment in select elements of adherence in cognitive training remain underexplored.

Another aspect of adherence is compliance with the prescribed cognitive training protocol, such as its schedule. Specifically, previous research has highlighted the importance of compliance with the intervention’s regimen to maximize engagement with the treatment paradigm (Chacko et al., 2014; Jones et al., 2020). However, a potential issue with attempting to comply with a dense cognitive training schedule is an increase in participant burden and/or decreased motivation, which may result in decreased perseverance and higher rates of participant attrition (Katz, Jaeggi, et al., 2018).

In a systematic review of the cognitive training literature, Tullo and Jaeggi (2022) aggregated the findings from studies that examined the effect of time between training sessions. The review concluded that the distribution of training sessions does not appear to have a significant impact on learning and transfer to targeted outcomes. In line with this observation, Schwaighofer et al.’s (2015) meta-analysis and Jaeggi et al.’s (2020) study did not reveal any significant relationship between the distribution of training sessions and outcome; while Wang et al. (2014) demonstrated benefits of training sessions that were spaced apart as compared to massed training. It is essential to note though that the range of training session distribution in existing cognitive training literature is relatively narrow, which could partially explain the lack of effects (Tullo & Jaeggi, 2022). To date, Wang et al. (2014) conducted the only empirical study that systematically varied the distribution of training sessions at a significant range, where one condition in their study dispersed training sessions from 2 to 20 calendar days. As such, it remains unclear whether spacing affects learning and engagement with the cognitive training paradigm.

Moreover, the extant literature examining spacing procedures is limited to fixed-effects designs; thus, it remains unclear whether providing participants with some autonomy in scheduling their training is beneficial for engagement with the training paradigm, and ultimately, learning. Beyond cognitive training, research has shown that autonomy and agency can lead to greater treatment adherence, and in turn, better health outcomes (see Entwistle et al., 2010). Autonomy and agency can be observed in a variety of healthcare interventions, such as choosing between different pharmacological treatment regimens (e.g., Williams et al., 1998) and making decisions about behavioral modifications (e.g., Sibley et al., 2022). Further characterizing the relationships between spacing and learning as well as consistency and learning are vital to cognitive training research, given that engagement and task-specific learning are precursors to improved outcomes (Donk et al., 2017; Jaeggi et al., 2011; Karbach et al., 2017; Katz et al., 2021; Ørskov et al., 2021; Redick, 2019; Wiemers et al., 2019).

### Aims of the Study

**Figure 1:**
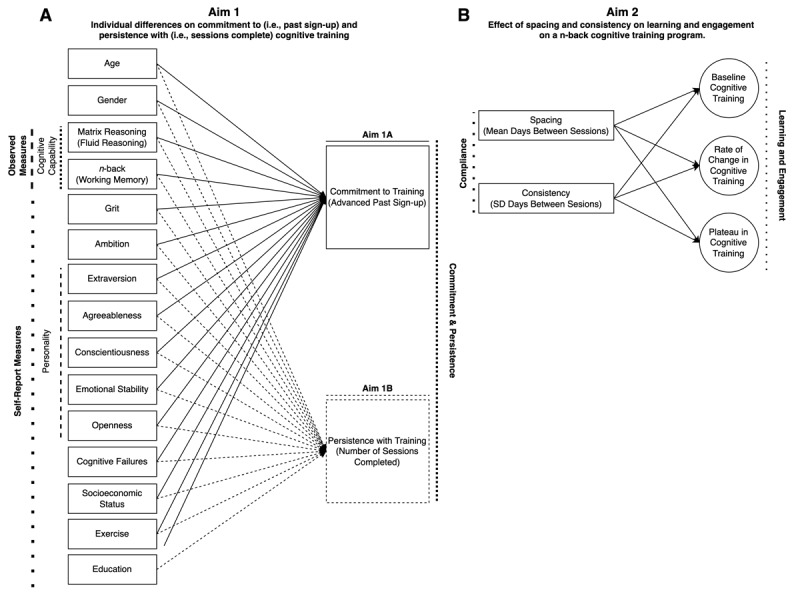
Study model illustrating the objectives of the study. **(A)** Investigation of individual difference factors influencing commitment to and persistence with cognitive training. **(B)** Examination of the impact of compliance factors on learning and engagement within the cognitive training paradigm.

Drawing upon the existing literature, this study aims to address two specific research gaps. Firstly, we will explore the association of various individual difference factors with commitment to (i.e., whether participants advanced past the sign-up portion of the study) and persistence with (i.e., the number of sessions completed) a 20-session cognitive training program (see Figure 1a). This aim is prompted by the evident scarcity of studies that examine these individual differences factors, such as age, gender, cognitive capability (i.e., fluid reasoning and working memory), grit, ambition, personality, self-perceived cognitive failures, socioeconomic status, exercise, and education. More specifically, previous research has produced inconsistent findings regarding the roles of age, personality traits, cognitive abilities, and commitment to and persistence with cognitive training (Cruz et al., 2014; Double & Birney, 2016; He et al., 2022; Turunen et al., 2019). Moreover, the effects of factors like self-reported grit, ambition, socioeconomic status, and exercise remain virtually unexplored and unknown. We anticipate that this aim will identify the extent to which certain individual difference factors indicate greater levels of perseverance to cognitive training paradigms and reconcile the inconsistency in the existing literature. The knowledge obtained here can inform the design of cognitive training programs, enhancing their efficacy by catering to the unique characteristics of participants (Katz et al., 2021).

Secondly, we aim to further understand treatment compliance by investigating the effect of spacing and consistency in cognitive training sessions on engagement and learning (see Figure 1b). Previous literature, although limited, offers mixed findings on the impact of session distribution (Jaeggi et al., 2020; Schwaighofer et al., 2015; Tullo & Jaeggi, 2022; Wang et al., 2014). Therefore, we will examine a broader range of training session distributions to gain a clearer understanding of their effect. Simultaneously, we will explore the potential benefits of autonomy in scheduling training sessions – an aspect that researchers have largely overlooked in cognitive training research but has demonstrated significance in other healthcare interventions (Entwistle et al., 2010; Williams et al., 1998). We anticipate that investigating the effect of spacing and consistency in cognitive training, if any, will provide valuable insights on optimizing training protocols for enhanced engagement and learning, contributing substantially to the existing body of knowledge on the mechanisms of cognitive training (Green et al., 2019).

## Method

### Participants

Participants were recruited for the study using widespread marketing via flyers and online advertisements. Between June 2021 and August 2022, we collected data from a sample of 4,775 adults aged between 18 and 93 years (*M* = 48.06, *SD* = 18.10) who signed up to participate in the study. Participant demographics show that 75% of the participants identified as female, 24% identified as male, and 1% indicated other or non-specific gender. The Institutional Review Boards at the University of California-Irvine and the University of California-Riverside approved the study procedure.

### Measures

#### Recollect – n-back training paradigm

**Figure 2:**
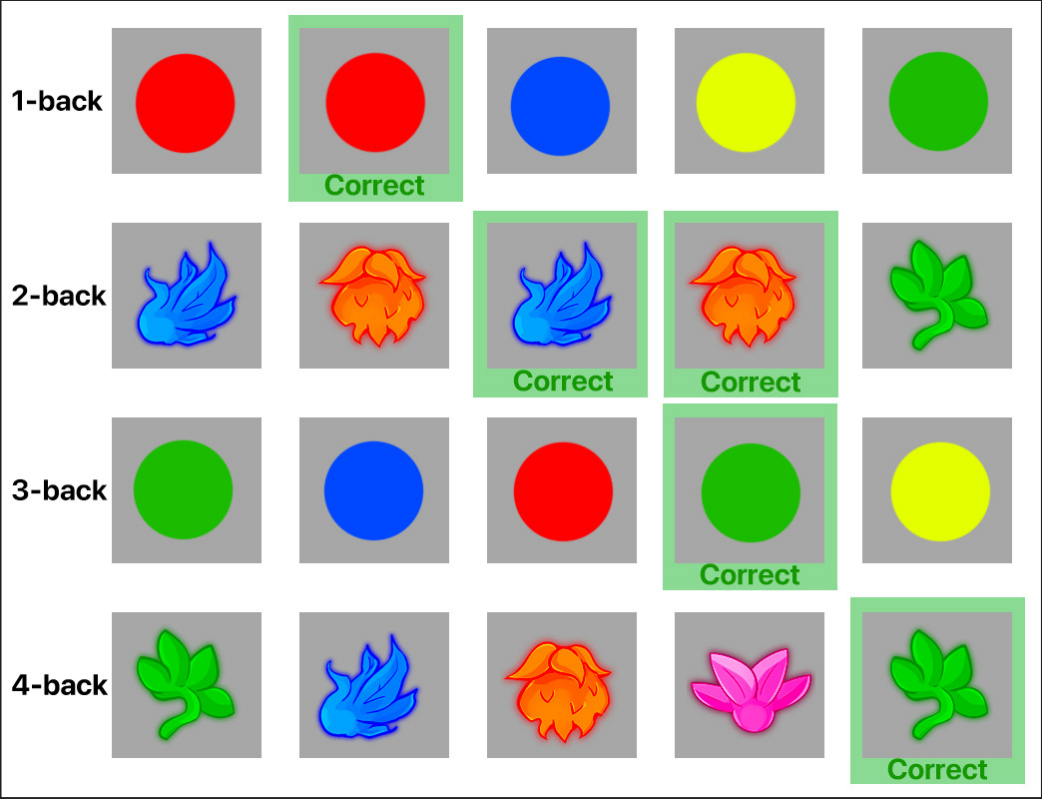
An illustration of the types of stimuli (i.e., colors and shapes) that were presented in the Recollect n-back training paradigm. The rows represent the correct responses for hits in conditions of 1-back to 4-back.

Participants trained on an app-based n-back paradigm that was developed by researchers at the University of California Riverside Brain Game Center called: “Recollect the Study”; available on Google Play; cf. Brain Game Center at UCR, 2019a; and Apple App Store; cf. Brain Game Center at UCR, 2019b). The application housing the n-back program was developed using the Unity platform, rendering it platform-independent and consequently accessible on both iOS and Android platforms. Recollect is a working memory training paradigm that has demonstrated efficacy in transfer to a variety of targeted outcomes ranging from proximal to distal domains (see Pahor et al., 2022). The n-back task assesses participants’ ability to identify stimuli that match those presented a given number of items back. In our task, participants are presented with a series of stimuli consisting of shapes and colors. They need to determine if the current stimulus matches the one presented *n* items back in the sequence (cf., Figure 2). To adjust the task difficulty, the n-back training task in this study adapts based on participants’ performance. Higher *n* levels require participants to remember items that occurred further back in the sequence, making the task more challenging. This adaptive approach ensures that the training is tailored to each individual participant’s abilities, rather than using a one-size-fits-all, standardized approach. Each session of the task is composed of multiple blocks, each lasting about three minutes, where each block includes approximately 60 or more *n* trials. The duration of the sessions varied between 20 and 30 minutes. The dependent variable of the Recollect – n-back training paradigm is the weighted average n-level achieved during a session. This is calculated by multiplying each n-level by the number of trials associated with that block and then dividing by the total number of trials.

The online cognitive training regimen affords the opportunity to investigate the predictive validity of individual differences factors in (i) committing to the cognitive training program (ii) persevering through the regimen, (iii) spacing between training sessions, and (iv) consistency in completing training sessions, across a large and representative population. Specifically, we asked whether individual difference factors such as age, gender, cognitive capability (i.e., measured by fluid reasoning and working memory), grit, ambition, personality, self-perceived cognitive failures, socioeconomic status, exercise, and education predicted whether participants committed to training, that is, whether they moved beyond the sign-up stage to the training phase. We also collected peripheral training data as measures of persistence. For instance, we collected data on the number of sessions completed as a measure of persistence. Participants could have completed a maximum of 20 sessions.

The online cognitive training regimen also affords the opportunity to examine compliance, that is, differences in spacing and consistency in cognitive training research. While participants were recommended to complete two training sessions per calendar day, participants were given the option to train at their leisure and availability. Therefore, we examined data illustrating spacing between training sessions, as measured by the average time between training sessions, and data characterizing consistency in training, as measured by the variance in time between training sessions.

#### Cognitive Capability Measures

##### Fluid Reasoning

A measure of fluid intelligence (cf., Pahor et al., 2019) was collected using the University of California Matrix Reasoning Task (UCMRT), which indicates an individual’s capability to solve non-verbal problems. Participants were asked to solve up to 23 problems within a 10-minute time limit. Specifically, each problem featured a 3 by 3 matrix with the lower right entry missing, and participants were instructed to select the answer option that best completes the matrix from a set of eight possible choices. Prior to assessment, participants were provided with sample problems and an opportunity to practice the task while receiving feedback. The main outcome measure was the percentage of correctly solved problems.

##### Working Memory

An untrained variant of the n-back task was administered to participants as a measure of working memory. The procedure and instructions for this task were similar to the cognitive training task; however, the stimuli provided to the participants consisted of animals or fruits and vegetables. Here we administered 1-back, 2-back, and 3-back levels to all participants in that sequence. Participants progressed to 4-back (and beyond) if they made no more than two errors on the previous level. Each level consisted of 30+*n* trials (i.e., the 3-back level would equate to 33 trials), nine of which were targets. Stimuli were displayed for 2.5 seconds with a 500 ms interval. To calculate accuracy for each level, hits were divided by the total number of hits, misses, and false alarms. The dependent variable was determined by averaging the z-scores of the accuracy levels for 2-back and 3-back.

#### Self-report Measures

##### Grit

Grit was assessed through Duckworth and Quinn’s (Duckworth & Quinn, 2009) questionnaire comprising eight statements, such as “I see things through to the end.” and represented indicators of perseverance and passion. Participants responded to each of the eight statements using a 5-point Likert scale, with possible responses ranging from “Not like me at all” to “Very much like me.” We calculated a composite score of all the questions, which served as the outcome variable for this measure.

##### Ambition

Ambition was assessed through Duckworth et al. ‘s (Duckworth et al., 2007) questionnaire comprising five statements, such as “I am a hard worker” and represented indicators of achievement seeking and success. Participants responded to each of the five statements using a 5-point Likert scale, with possible responses ranging from “Not like me at all” to “Very much like me.” We calculated a composite score of all the questions, which served as the outcome variable for this measure.

##### Personality

We collected participant self-report ratings on the big five personality traits (i.e., Emotional Stability, Extraversion, Openness, Agreeableness, and Conscientiousness) using the 40-item Mini-Markers questionnaires (Saucier, 1994). Each personality trait was measured using a series of eight adjective-based items. Participants responded with the extent to which each item was representative of their character on a 5-point Likert scale. For instance, when assessing the personality trait of Extraversion, one of the items used was ‘Bold.’ Consequently, participants were asked to rate their agreement on a scale from 1 (i.e., extremely inaccurate) to 5 (i.e., extremely accurate). The composite score for all eight items was used as the outcome variable for each personality trait.

##### Cognitive Failure Questionnaire

The Cognitive Failure Questionnaire (CFQ) by Broadbent et al. (Broadbent et al., 1982) was used to gather information about the self-perceived memory capabilities of participants. Participants were asked to indicate the frequency of instances where they experienced memory lapses, such as forgetting people’s names, appointments, and leaving appliances turned on. The response for each of the eight items was on a five-point Likert scale, where a low score indicated the participant never experienced the memory lapse and a high score indicated the participant experienced the event very often (e.g., “*Do you find that you forget whether you’ve turned off a light, or the stove, or locked the door?”*). We calculated a composite score of all the items, which served as the outcome variable for this measure.

##### Socio-economic Status

We assessed participants’ subjective Socio-economic Status (SES) via the MacArthur Scale of Subjective Social Status (cf., Adler et al., 2000). The measure has demonstrated more reliable and robust associations with health, well-being and status compared to traditional and direct measure of SES, such as income (cf., Garza et al., 2017; Singh-Manoux et al., 2005). We presented two ladder pictures, where the first ladder represented community status and the second ladder represented money, education, and job status. The participant rated where they were best represented on a given ladder rung; that is, the top of the ladder was coded as 10, indicating the highest SES, while the bottom was coded as 1, indicating the lowest SES. The resulting scores for both ladders were added together to determine the overall self-reported socioeconomic status level.

##### Exercise

We collected the participant’s fitness routine as an indicator of discipline to health. Participants were asked to report their weekly activity time using a 6-point Likert scale, where a rating of 6 indicated over four hours of activity per week, and a rating of 1 indicated less than 30 minutes of activity.

##### Education

Participants were asked to indicate their highest degree achieved using the following response options: less than a high school degree, high school or GED equivalent degree, associate degree, bachelor’s degree, master’s degree, professional degree (e.g., M.D., J.D., D.D.S., etc.), and doctorate degree (e.g., Ph.D., Psy.D., Ed.D., etc.). Based on these responses, participants were grouped into three categories: (i) those with an associate degree or a high school degree or less, (ii) those with a bachelor’s degree, and (iii) those with an advanced degree (i.e., master’s, doctorate, and professional degree). From here, education was dummy coded into two variables. The first variable, Education (Secondary), included participants with an associate degree, high school degree, or less as the target group. The second variable, Education (Advanced), included participants with a master’s, doctorate, or professional degree as the target group. Consequently, individuals with a bachelor’s degree served as the reference group in subsequent analyses, with these two dummy coded variables included.

### Procedure

The study involved administering a series of measures and questionnaires to participants using Qualtrics Software. The administered measures included demographic questionnaires, cognitive measures, and self-report measures. Participants completed these measures and training sessions on their electronic devices.

Prior to the training phase, participants underwent cognitive assessments measuring fluid reasoning and working memory. During the training phase, participants were instructed to complete 20 training sessions, with a requirement to complete two sessions each calendar day. However, the software did not restrict participants from modifying their training schedule. Consequently, participants exhibited variations in the spacing and consistency of their training sessions, despite the maximum limit of two sessions per 24-hour rotation and a total of 20 sessions.

### Data analysis plan

We analyzed the data using R version 4.2.2 and R Studio Version 2022.12.0+353. Firstly, we conducted univariate outlier analyses to identify and remove scores beyond 3.5 standard deviations above or below the mean. To examine the aim of identifying individual difference factors that predict commitment to cognitive training, we performed preliminary t-tests to assess the association between predictor variables and the dependent variables. After the preliminary analyses, we used logistic regression to examine the predictive validity of several individual difference factors, including age, gender, fluid reasoning, working memory (i.e., cognitive capability), grit, ambition, personality, self-perceived cognitive failures, socioeconomic status, exercise, and education, in relation to advancing or participating in cognitive training sessions. Before conducting the logistic regression analyses, we screened the data for multicollinearity and treated missing data with casewise deletion.

**Figure 3:**
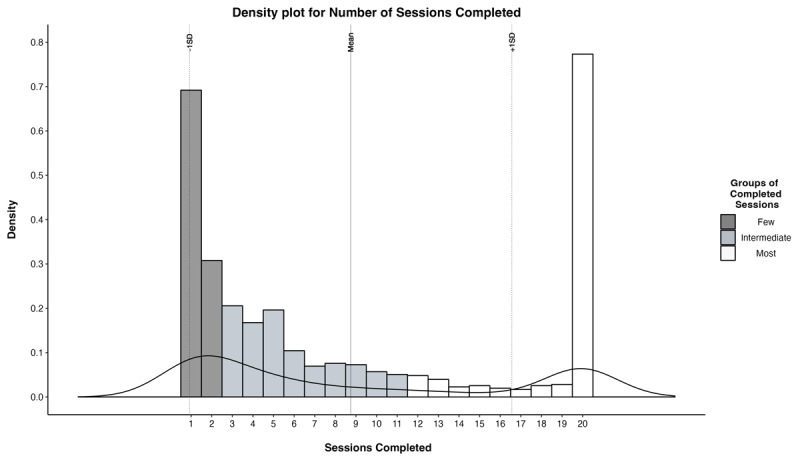
The frequency of completed sessions on the cognitive training paradigm for participants who progressed to the training phase of the study is presented through a histogram and a density plot. The density plot represents the relative likelihood of the value occurring using the probability and is displayed using the smoothed curve as an estimate of the underlying distribution of the sessions completed variable (y-axis). The histogram bars display the frequency distribution of completed sessions and are color-coded based on naturally occurring groups divided into thirds: dark grey for participants who completed a ‘Few’ number of sessions, light grey for participants who completed an ‘Intermediate’ number of sessions, and white for participants who completed the ‘Most’ number of sessions.

Next, to address the study aim of identifying individual difference factors that predict persistence with cognitive training, we conducted multinomial regression analysis to assess the predictive validity of these factors across three levels of training progress. Due to the bimodal distribution in the sample size, we divided the participants into three groups based on the number of sessions completed (out of a maximum of twenty). Specifically, we categorized a group as “Few” completions if they completed the first two sessions, representing the bottom 33.3% percentile rank. The “Intermediate” group consisted of participants who completed the first half of the remaining sessions beyond initial training (sessions 3–11), representing percentile rank scores between 33.4% and 66.5%. Finally, the “Most” group included participants who completed the second half of the remaining sessions (sessions 12–20), with percentile rank scores of 66.6% and above (refer to Figure 3). Table 1 provides information on the count per group, the mean number of sessions completed, the standard deviation of sessions completed, as well as the range in sessions completed, as indicated by the minimum and maximum values.

**Table 1:** Cognitive training sessions completed by group.


GROUP	n	MEAN	SD	MIN	MAX

Few	354	1.31	0.46	1	2

Intermediate	316	5.77	2.43	3	11

Most	353	18.86	2.41	12	20

**Total**	**1,023**	**8.74**	**7.82**	**1**	**20**


*Note*: Out of the 4,775 participants that signed up for the study and completed self-reported measures, only 1,023 completed at least one training session.

Lastly, we focused on the subsample of participants who completed all 20 training sessions to examine the aim associated with characterizing the role of spacing and consistency on learning and engagement with a cognitive training paradigm. In this analysis, we explored the relationship between compliance and engagement, beyond individual difference factors. To analyze our data, we utilized a latent growth curve model (LGCM) with structural equation modeling in R studio, using the lavaan package version 0.6–12 (Rosseel, 2012). Before the main analyses, we removed univariate outliers for the first two training sessions to account for participants who may not have understood the task parameters based on unlikely scores.

First, we assessed the fit of the repeated measures data of the training paradigm to different models, including an intercept-only model, a linear trend, and the hypothesized logarithmic trend that represents a typical learning curve (Guye et al., 2017; Ørskov et al., 2021; Orylska et al., 2019). Next, we introduced predictors of compliance measures, such as spacing (average time between sessions) and consistency of training sessions (standard deviation of time between sessions), along with observed measures of cognitive capability (fluid reasoning and working memory) and demographic variables (age and gender). We only included participants who completed all twenty training sessions, ensuring no missing training data.

## Results

### Individual difference factors and commitment to training

First, we examined differences in individual-level factors based on naturally occurring groups of participants: those who progressed to the cognitive training portion of the study and those who dropped out of the study prior to the cognitive training phase. Table 2 presents observed data for each variable, including the mean, standard deviation, and effect size indicating the magnitude of group differences. Additionally, the table presents the corresponding *t*-test results and Cohen’s *d* effect size (i.e., with Bonferroni-corrected confidence intervals) for the individual difference factor between participants who advanced to training and those who did not. As presented in the table, there was a small difference in ratings of grit: *t*(4,520) = 2.85, *p* = .004; *d* = 0.08, 99.6% CI [0.00, 0.22] and the personality trait of conscientiousness: *t*(4,506) = 3.08, *p* = .002; *d* = 0.09, 99.6% CI [0.00, 0.23] between the two naturally occurring groups, where those that advanced to training demonstrated higher ratings of conscientiousness compared to those that did not advance to training. Additionally, there were significant differences in the categorical variables of education (i.e., highest degree attained). Here, there were more participants with an advanced degree (i.e., master’s, doctorate, or professional degree) that completed at least one more training session: χ^2^(1) = 5.66, *p* = .017; Cramer’s V = .04, 99.6% CI [.02, .09]. Similarly, there were fewer participants with a high-school degree, equivalent, or less that completed at least one more training session than those with a post-secondary degree: χ^2^(1) = 5.66, *p* = .017; Cramer’s V = .04, 99.6% CI [.02, .09]. Nevertheless, while these individual difference factors reached statistical significance, their effect size were small and negligible, and as such the test statistics may have been inflated due to the large sample (i.e., Type 1 error). There were no other statistically detectable group differences after controlling for family-wise error.

**Table 2:** Group differences in predictors between participants that committed to training versus those that did not commit to training.


VARIABLE	COUNT	ADVANCED TO TRAINING			EFFECT SIZE; *STATISTICAL TEST*

		NO	YES	TOTAL	

		n = 3,752	n = 1,023	N = 4,775	

Age	4,622	48.23(18.25)	47.38(17.36)	**48.07(18.09)**	*d* = –0.04, 99.69% CI [–0.16, 0.06]; *t*(4,620) = –1.24, *p* = .213

Matrix Reasoning	1,877	50.50(19.07)	50.69(19.38	**50.58(19.20)**	*d* = 0.01, 99.69% CI [–0.13, 0.15]; *t*(1,875) = 0.21, *p* = .833

Working Memory	2,793	0(1.29)	0.02(1.27)	**0.01(1.28)**	*d* = 0.01, 99.69% CI [–0.10, 0.13]; *t*(2,791) = 0.37, *p* = .709

Grit	4,522	26.7(5.31)	27.26(5.11)	**26.8(5.28)**	*d* = 0.08, 99.69% CI [0.00, 0.22]; *t*(4,520) = 2.85, *p* = .004

Ambition	4,522	17.19(3.95)	17.26(3.92)	**17.2(3.95)**	*d* = 0.01, 99.69% CI [–0.09, 0.13]; *t*(4,520) = 0.46, *p* = .649

Extraversion	4,508	25.28(6.41)	24.79(6.51)	**25.18(6.43)**	*d* = –0.06, 99.69% CI [–0.19, 0.04]; *t*(4,506) = –2.03, *p* = .043

Agreeableness	4,508	33.22(4.65)	32.96(4.73)	**33.17(4.66)**	*d* = –0.05, 99.69% CI [–0.17, 0.05]; *t*(4,506) = –1.52, *p* = .129

Conscientiousness	4,508	30.37(5.6)	31.02(5.49)	**30.49(5.59)**	*d* = 0.09, 99.69% CI [0.00, 0.23]; *t*(4,506) = 3.08, *p* = .002*

Emotional Stability	4,508	27.25(5.82)	27.75(5.89)	**27.34(5.84)**	*d* = 0.07, 99.69% CI [–0.02, 0.20]; *t*(4,506) = 2.28, *p* = .022

Openness	4,508	31(4.83)	31.16(4.72)	**31.03(4.81)**	*d* = 0.03, 99.69% CI [–0.08, 0.14]; *t*(4,506) = 0.86, *p* = .391

Cognitive Failures	4,495	24.48(5.74)	24.05(5.44)	**24.4(5.69)**	*d* = –0.06, 99.69% CI [–0.19, 0.04]; *t*(4,493) = –2.03, *p* = .042

SES	4,558	9.59(3.35)	9.46(3.22)	**9.56(3.33)**	*d* = –0.03, 99.69% CI [–0.15, 0.07]; *t*(4,558) = –1.04, *p* = .299

Exercise	4,597	2.31(1.69)	2.4(1.64)	**2.33(1.68)**	*d* = 0.04, 99.69% CI [–0.06, 0.16]; *t*(4,595) = 1.38, *p* = .168

Gender (Female)	4,622				Cramer’s V = .01, 99.69% CI [.01, .05]; χ^2^(1) = 0.21, *p* = .647

*NO*		25 % (930)	26 % (223)	**25 % (1,153)**	

*YES*		75 % (2,802)	74 % (646)	**75 % (3,448)**	

*Education (Advanced)*	3,642				Cramer’s V = .04, 99.6% CI [.02, .09]; χ^2^(1) = 5.66, *p* = .017*

*NO*		50 % (1403)	45 % (369)	**49 % (1772)**	

*YES*		50 % (1419)	55 % (451)	**51 % (1870)**	

*Education (Secondary)*	3,642				Cramer’s V = .04, 99.69% CI [.02, .09]; χ^2^(1) = 6.16, *p* = .013*

*NO*		78 % (2214)	82 % (676)	**79 % (2890)**	

*YES*		22 % (608)	18 % (144)	**21 % (752)**	


*Note*: Means and (Standard Deviations) are presented across continuous individual difference factors investigated in the study and percentages and count statistics are reported for categorical variables. * Denotes a significant difference after Bonferroni correction after 15 comparisons. Categorical variables of Education (Advanced) were grouped by those that have a master’s, doctorate, or professional degree as the highest degree attained (YES) compared to those that do not (NO); Education (Secondary) were grouped by those that have less than high-school, high-school or GED as the highest degree attained (YES) compared to those that do not (NO).

A logistic regression analysis was conducted to examine the relationship between observed predictor variables and whether participants progressed through to the cognitive training portion of the study. The model included age, gender (i.e., with individuals that identified as female as the target variable and individuals that did not identify as females, that is, male and other gender non-specified as the reference category), matrix reasoning accuracy, and working memory accuracy as the predictor variables. Given the wide age range, interaction estimates between age and matrix reasoning and working memory were added to the model. The results suggest that the model did not provide a good fit to the data: χ^2^(7) = 11.83, *p* = .11, McFadden’s pseudo *R*^2^ = 0.01, with 1,603 observations after casewise deletion for missing data (see Table 3).

**Table 3:** Logistic regression summary for predicting commitment to training by model.


NAME	b	SE	OR	OR 95% CI	*p*

(Intercept)	–0.36	0.48	0.7	[0.27, 1.78]	0.452

Age	0	0.01	1	[0.99, 1.02]	0.592

Female (YES)	–0.19	0.12	0.83	[0.66, 1.05]	0.117

Matrix Reasoning	0.01	0.01	1.01	[0.99, 1.03]	0.299

n-back	–0.2	0.13	0.82	[0.63, 1.06]	0.133

Age × Matrix Reasoning	0	0	1	[0.99, 1.00]	0.236

Age × n-back	0	0	1	[1.00, 1.01]	0.05

**Observed measures model: χ^2^(7) = 11.83, *p* = .11, McFadden’s *R*^2^ = 0.01; N = 1,603**					

(Intercept)	–1.42	0.87	0.24	[0.04, 1.34]	0.104

Age	0	0.01	1	[0.97, 1.03]	0.985

Female (YES)	–0.01	0.1	0.99	[0.82, 1.21]	0.934

Grit	0.01	0.01	1.01	[0.98, 1.03]	0.585

Ambition	–0.02	0.01	0.98	[0.96, 1.01]	0.198

Extraversion	–0.01	0.01	0.99	[0.97, 1.00]	0.038*

Agreeableness	–0.02	0.01	0.98	[0.96, 0.99]	0.011*

Conscientiousness	0.01	0.01	1.01	[0.99, 1.03]	0.16

Emotional Stability	0.02	0.01	1.02	[1.00, 1.03]	0.043*

Openness	0.01	0.01	1.01	[0.99, 1.03]	0.169

Cognitive Failures	0	0.02	1	[0.96, 1.04]	0.945

SES	–0.01	0.04	0.99	[0.92, 1.07]	0.778

Exercise	0.06	0.07	1.06	[0.92, 1.22]	0.451

Education (Secondary)	–0.32	0.33	0.73	[0.38, 1.39]	0.339

Education (Advanced)	0.46	0.29	1.59	[0.91, 2.79]	0.106

Age × Cognitive Failures	0	0	1	[0.99, 1.00]	0.971

Age × SES	0	0	1	[0.99, 1.00]	0.567

Age × Exercise	0	0	1	[0.99, 1.00]	0.603

Age × Education (Secondary)	0	0.01	1	[0.99, 1.02]	0.5

Age × Education (Advanced)	–0.01	0.01	0.99	[0.98, 1.00]	0.259

**Self-report measures model: χ^2^(22) = 11.83, *p* = .11, McFadden’s *R*^2^ = 0.02; N = 3,295**					


*Note*: Unstandardized beta coefficients (b), the standard error (SE), Odds Ratio (OR), the 95% confidence intervals [lower limit, upper limit], and p-value are presented by model (i.e., first logistic regression with observed predictors on top and second logistic regression with self-report measures on the bottom. Model chi-square test, McFadden’s pseudo *R^2^*, and sample size are also presented.

A second logistic regression analysis was conducted to determine the factors that predict advancing to the training phase of the study. The second model included self-report predictor variables consisting of the GRIT questionnaire, Ambition questionnaire, all 5 personality indicators (i.e., extraversion, agreeableness, conscientiousness, emotional stability, and openness), cognitive failures questionnaire, SES, exercise, and education (i.e., education [secondary] and education [advanced]). To account for the wide age range, interaction estimates between age and cognitive failures, SES, exercise, and education were also included in the model. The overall second model was significant: χ^2^(22) = 34.44, *p* = .016, consisting of 3,295 observations after casewise deletion due to missing data; however, the self-report predictors explained only a small portion of the variance in whether participants advanced to the training phase (McFadden’s pseudo *R*^2^ = .01). While the model and several predictors were statistically tenable, the small overall model effect size and small effects of predictors suggest that the self-report individual difference factors had negligible effects on the likelihood of participants progressing to the cognitive training portion of the study (see Table 3).

### Persistence with training

Next, we conducted two multinomial regression analyses to predict differences between three groups categorized based on the number of cognitive training sessions completed (i.e., few, intermediate, and most; cf., Table 1). The analyses aimed to examine the impact of (i) behavioral measures and (ii) self-report questionnaires on these groups. More specifically, we used the *Few* group as the reference group, and as such compared differences between the most group and the *Few* group and between the *Intermediate* group and the *Few* group.

The first multinomial regression model included age, gender, matrix reasoning accuracy, and n-back accuracy as the predictor variables, and consisted of 674 complete observations out of the 1,022 participants that advanced to the cognitive training stage. Considering the extensive age range, interaction estimates between age and all predictors were incorporated into the model. This model with observed predictors, was predictive of the group classification χ^2^(8) = 27.34, *p* = .007; albeit, with a small effect size: McFadden’s pseudo *R*^2^ = .02. The model correctly classified 21.72%, performing below chance level (i.e., 33.3%). More specifically, 67.13% of the participants in the Most group were successfully classified above chance; however, participants in the Intermediate and Few groups were classified below chance level at 29.18% and 28.81%, respectively. No observed predictor by group difference effect was statistically tenable (see Table 4).

**Table 4:** Multinomial regression summary of predictors for completed sessions by model.


NAME	b	SE	OR	OR 95% CI	*p*

(Intercept) x Intermediate	0.5	1.09	1.65	[0.20, 13.85]	0.645

(Intercept) x Most	0.06	1.1	1.06	[0.12, 9.14]	0.956

Age x Intermediate	0.01	0.02	1.01	[0.97, 1.05]	0.712

Age x Most	0.02	0.02	1.02	[0.98, 1.06]	0.384

Matrix Reasoning x Intermediate	0.01	0.02	1.01	[0.97, 1.05]	0.505

Matrix Reasoning x Most	0.01	0.02	1.01	[0.97, 1.05]	0.622

n-back x Intermediate	–0.34	0.3	0.71	[0.39, 1.29]	0.259

n-back x Most	–0.19	0.31	0.83	[0.45, 1.51]	0.535

Female x Intermediate	0.03	0.28	1.03	[0.59, 1.79]	0.916

Female x Most	–0.29	0.27	0.75	[0.44, 1.27]	0.278

Age x Matrix Reasoning x Intermediate	0	0	1	[0.97, 1.05]	0.554

Age x Matrix Reasoning x Most	0	0	1	[0.98, 1.06]	0.982

Age x n-back x Intermediate	0	0.01	1	[0.97, 1.05]	0.518

Age x n-back x Most	0	0.01	1	[0.98, 1.06]	0.5

**Observed measures model: χ^2^(16) = 28.24, *p* = .013, McFadden’s pseudo *R*^2^ = 0.02; N = 674**					

(Intercept) x Intermediate	–2.69	2.03	0.07	[0.001, 3.61]	0.184

(Intercept) x Most	1.22	2.03	3.39	[0.06, 181.30]	0.548

Age x Intermediate	0.04	0.03	1.04	[0.98, 1.12]	0.212

Age x Most	0	0.03	1	[0.93, 1.06]	0.899

Grit x Intermediate	0.01	0.03	1.01	[0.96, 1.06]	0.769

Grit x Most	0	0.03	1	[0.95, 1.05]	0.999

Ambition x Intermediate	0.02	0.03	1.02	[0.96, 1.08]	0.566

Ambition x Most	–0.03	0.03	0.97	[0.92, 1.03]	0.338

Extraversion x Intermediate	–0.03	0.02	0.97	[0.95, 1.00]	0.098

Extraversion x Most	–0.03	0.01	0.97	[0.94, 1.00]	0.032*

Agreeableness x Intermediate	0.03	0.02	1.03	[0.99, 1.08]	0.119

Agreeableness x Most	0.01	0.02	1.01	[0.97, 1.05]	0.584

Emotional Stability x Intermediate	0	0.02	1	[0.97, 1.04]	0.791

Emotional Stability x Most	0.01	0.02	1.01	[0.97, 1.04]	0.671

Openness x Intermediate	–0.05	0.02	0.95	[0.91, 0.99]	0.021*

Openness x Most	–0.01	0.02	0.99	[0.95, 1.03]	0.491

Conscientiousness x Intermediate	0.04	0.02	1.04	[1.00, 1.09]	0.072

Conscientiousness x Most	0.02	0.02	1.02	[0.98, 1.07]	0.279

Cognitive Failures x Intermediate	0	0.05	1	[0.91, 1.11]	0.92

Cognitive Failures x Most	–0.14	0.05	0.87	[0.79, 0.96]	0.008*

SES x Intermediate	0.07	0.09	1.07	[0.89, 1.29]	0.448

SES x Most	0.08	0.1	1.08	[0.89, 1.31]	0.417

Exercise x Intermediate	0.23	0.17	1.26	[0.90, 1.76]	0.171

Exercise x Most	0.15	0.17	1.16	[0.83, 1.63]	0.389

Education (Secondary) x Intermediate	1.06	0.74	2.89	[0.68, 12.24]	0.149

Education (Secondary) x Most	1.47	0.81	4.33	[0.89, 21.02]	0.069

Education (Advanced) x Intermediate	0.55	0.64	1.74	[0.49, 6.11]	0.391

Education (Advanced) x Most	1.46	0.67	4.32	[1.16, 16.12]	0.029*

Age x Cognitive Failures x Intermediate	0	0	1	[0.98, 1.12]	0.952

Age x Cognitive Failures x Most	0	0	1	[0.93, 1.06]	0.017*

Age x SES x Intermediate	0	0	1	[0.98, 1.12]	0.498

Age x SES x Most	0	0	1	[0.93, 1.06]	0.296

Age x Exercise x Intermediate	–0.01	0	0.99	[0.98, 1.12]	0.092

Age x Exercise x Most	0	0	1	[0.93, 1.06]	0.572

Age x Education (Secondary) x Intermediate	–0.03	0.02	0.97	[0.95, 1.12]	0.092

Age x Education (Secondary) x Most	–0.03	0.02	0.97	[0.93, 1.07]	0.038*

Age x Education (Advanced) x Intermediate	–0.02	0.01	0.98	[0.96, 1.12]	0.208

Age x Education (Advanced) x Most	–0.03	0.01	0.97	[0.93, 1.06]	0.028*

**Self-report measures model: χ^2^(24) = 61.93, p = .004, McFadden’s pseudo *R*^2^ = 0.04; N = 755**					


*Note*: Unstandardized beta coefficients (b), the standard error (SE), Odds Ratio (OR), the 95% confidence intervals [lower limit, upper limit], and p-value are presented by model (i.e., first logistic regression with observed predictors on top and second logistic regression with self-report measures on the bottom. Model chi-square test, McFadden’s pseudo *R^2^*, and sample size are also presented. Socioeconomic Status (SES).

The second multinomial model with self-report predictors, consisted of 755 complete observations out of the 1,022 participants that advanced to the cognitive training stage. This model with self-report predictors was also predictive of the group classification χ^2^(24) = 61.93, *p* = .004; with once again a small effect size: McFadden’s pseudo *R*^2^ = .04 (see Table 4). The model correctly classified 43.44%, performing above chance level (i.e., 33.3%). More specifically, 67.13% of the participants were correctly classified in the *Most* group. However, participants in the *Few* group and participants in the *Intermediate* group were classified below chance level at 28.81 and 29.18%, respectively. Moreover, there was an effect of Education (Advanced) between *Most* and *Few* groups, an having attained an advanced degree (i.e., master’s, doctorate, or professional) was associated with a greater likelihood of completing most of the training sessions compared to just a few of the training sessions: (*b* = 1.46, *OR* = 4.32, 95% CI [1.16, 16.12], *p* = .002), compared to those that had less than an advanced degree. Similarly, there was an effect of cognitive failures between *Most* and *Few* groups, where an increase in cognitive failures was associated with a greater likelihood of completing just a few of the training sessions compared to most of the training sessions: (*b* = –0.14, *OR* = 0.87, 95% CI [0.79, 0.96], *p* = .008). While there were other predictors that reached statistical significance, their effect size straddled 1.0, and as such, did not have a meaningful effect on the number of training sessions.

As a follow-up to the analysis on persistence, we examined the predictive validity of individual difference factors between those that completed all 20 sessions and those that completed most but not all the training sessions (i.e., 12 to 19 sessions). Given the small effects between the *Most and Intermediate groups*, we conducted two additional logistic regression analyses to examine what cognitive measures (i.e., fluid reasoning, and working memory) and demographic variables (i.e., age, gender), as well as self-report measures (i.e., grit questionnaire, ambition questionnaire, all 5 personality indicators cognitive failures questionnaire, SES, exercise, and education predict participants’ persistence in completing all 20 sessions.

The overall model for behavioral measures was not significant: χ^2^(6) = 8.74, *p* = .189; McFadden’s pseudo *R*^2^ = .03, consisting of 318 participants that completed all 20 sessions and those that completed most but not all the training sessions (i.e., 12 to 19 sessions). There were no statistically detectable predictors in the model (see Table 5).

**Table 5:** Logistic regression summary of predictors for most completed sessions by model.


NAME	b	SE	OR	OR 95% CI	*p*

(Intercept)	–0.32	1.34	0.72	[0.05, 10.20]	0.81

Age	0.02	0.03	1.03	[0.98, 1.08]	0.33

Female (YES)	0.05	0.31	1.05	[0.57, 1.91]	0.865

Matrix Reasoning	0.01	0.02	1.01	[0.96, 1.06]	0.737

n-back	0.06	0.34	1.06	[0.55, 2.08]	0.853

Age x Matrix Reasoning	0	0	1	[0.99, 1.00]	0.998

Age x n-back	0	0.01	1	[0.99, 1.01]	0.935

**Observed measures model: χ^2^(6) = 8.74, *p* = .189, McFadden’s pseudo *R*^2^ = 0.03; N = 318**					

(Intercept)	6.26	3.69	525.54	[0.41, 867756.75]	0.09

Age	–0.04	0.06	0.96	[0.85, 1.08]	0.485

Grit	0.04	0.05	1.04	[0.95, 1.15]	0.37

Ambition	–0.04	0.05	0.96	[0.87, 1.06]	0.433

Extraversion	–0.03	0.03	0.97	[0.92, 1.02]	0.223

Agreeableness	–0.03	0.04	0.97	[0.90, 1.04]	0.378

Conscientiousness	0.02	0.04	1.02	[0.95, 1.10]	0.582

Neurotic	–0.02	0.03	0.98	[0.92, 1.04]	0.554

Openness	–0.02	0.04	0.98	[0.91, 1.05]	0.63

Cognitive Failures	–0.06	0.09	0.94	[0.79, 1.12]	0.477

SES	–0.14	0.15	0.87	[0.65, 1.18]	0.362

Exercise	–0.04	0.29	0.96	[0.54, 1.69]	0.877

Education (Secondary)	0.21	1.45	1.24	[0.07, 21.62]	0.883

Education (Advanced)	–2.01	1.2	0.13	[0.01, 1.31]	0.095

Age x Cognitive Failures	0	0	1	[1.00, 1.00]	0.866

Age x SES	0	0	1	[1.00, 1.01]	0.269

Age x Exercise	0	0.01	1	[0.99, 1.01]	0.709

Age x Education (Secondary)	–0.02	0.03	0.98	[0.93, 1.04]	0.548

Age x Education (Advanced)	0.04	0.02	1.04	[0.99, 1.09]	0.112

**Self-report measures model: χ^2^(18) = 26.02, p = .099, McFadden’s pseudo *R*^2^ = 0.13; N = 309**					


*Note*: Unstandardized beta coefficients (b), the standard error (SE), Odds Ratio (OR), the 95% confidence intervals [lower limit, upper limit], and p-value are presented by model (i.e., first logistic regression with observed predictors on top and second logistic regression with self-report measures on the bottom. Model chi-square test, McFadden’s pseudo *R^2^*, and sample size are also presented.

The overall model for self-report measures was also not statistically significant: χ^2^(18) = 26.02, *p* = .099; McFadden’s pseudo *R*^2^ = .13, consisting of 309 complete observations from participants that completed all 20 sessions and those that completed most but not all the training sessions (i.e., 12 to 19 sessions). Nevertheless, there were no statistically detectable predictors on group membership (see Table 5).

### Spacing and Consistency

We examined the role of spacing and consistency in separate models that predicted engagement on the cognitive training paradigm, as measured by repeated measures on the Recollect training task. To best answer this research question, we limited the sample to participants that completed all 20 training sessions. Additionally, we removed univariate outliers in initial training days, as well as spacing and consistency. We also removed cases with extreme scores on spacing and consistency, as indicated by z-scores ranging beyond –3.5 and +3.5 z-scores. Here, 11 participants were removed from the main analysis. In total, 263 participants were included in the following models.

First, we examined the bivariate relationship between spacing and consistency and found that there was a strong and robust correlation between the two variables of interest: *r*(261) = .85, 95% CI [.81, .88]. As such, spacing and consistency were included as predictors in separate models on account of multicollinearity between the two variables of interest.

**Figure 4:**
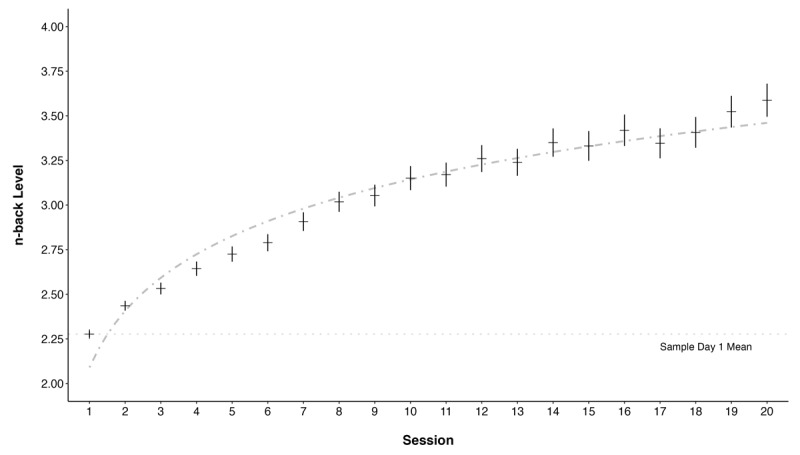
Aggregated performance trajectory across the entire sample (N = 263). Means and standard errors for each training session’s n-back level are illustrated.

As a precursor to the conditional LGCM, we examined the fit of the engagement data on the Recollect task across the 20 training sessions to an intercept-only model, a linear model, and a logarithmic model (see Bauer & Curran, 2019). The results for the intercept-only, baseline model, demonstrated poor fit indices. We then fit the data to a linear trend model, which also demonstrated poor fit indices; yet, demonstrated significance on the scaled chi-squared difference test comparing the value-added of the linear slope as a latent variable beyond the intercept-only model: Δχ^2^(3) = 1396.2, *p* < .001. Similarly, the addition of the logarithmic (i.e., base 2) latent variable demonstrated significance on the scaled chi-squared difference test in comparison with the linear model: Δχ^2^(4) = 579.52, *p* < .001. Moreover, the logarithmic model demonstrated adequate fit (see supplemental table 1 for fit indices presented across the baseline, linear, and logarithmic functions). The unconditional logarithmic model demonstrated a statistically detectable covariance between the intercept and the log variable, as well as the slope and the log variable. The statistically significant covariance suggests that (i) participants’ baseline capabilities are related to the plateau of performance and (ii) the initial rate of change in learning is also related to the plateau of performance. There was no statistically significant covariance between the intercept and the slope, indicating that baseline capabilities are not related to the initial increase in performance (see supplemental table 2 for means, variances, and covariance for all latent variables and between unconditional and conditional models). Figure 4 represents repeated measures training data for the entire sample.

Following the measurement model, we introduced spacing and consistency, in separate conditional models, along with observed predictors of age, gender, fluid reasoning, and working memory. The first conditional model with spacing, age, gender, fluid reasoning, and working memory predictors, as well as the interaction between age and fluid reasoning and working memory. The conditional model also demonstrated adequate fit indices, as well as significant relationships between the intercept and the log, and the slope and the log variables. Similarly, there was no significant relationship between the intercept and slope variables.

**Figure 5:**
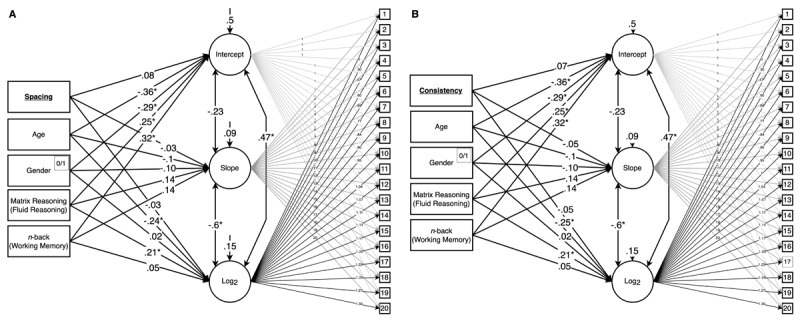
Statistical models of latent growth curve models are divided into two aspects: spacing (**A**; left) and consistency (**B**; right) in engagement with the working memory paradigm. The left-most rectangles in each model represent the model predictors, which were used to investigate their relationship with learning curve attributes, including the intercept, slope, and log base 2 portions of the logarithmic curve. The rightmost squares represent performance across sessions 1 through 20, which contribute to the latent variable curve attributes.

The model examined the relationship between the intercept factor, which represented the baseline performance in Recollect, and several predictors. Spacing was not a statistically detectable predictor of baseline performance above and beyond other predictors in the model (Ŷ_S-INT_ = .08, *p* = .310). Nevertheless, the results showed that age, gender, fluid reasoning capability, and working memory were statistically significant predictors of the intercept factor. Specifically, older participants had lower baseline scores than younger participants (Ŷ_age-INT_ = –.36, *p* < .001), females demonstrated lower baseline performance than those that did not identify as female (Ŷ_gender-INT_ = –.29, *p* = .033), participants with higher matrix reasoning accuracy scores had higher baseline training capability compared to those with lower scores (Ŷ_FR-INT_ = .25, *p* < .001), and similarly, participants with higher working memory capability had higher initial training performance (Ŷ_WM-INT_ = .32, *p* < .001). The analysis did not detect any statistically significant predictors on the linear slope of the training data (age: Ŷ_age-SLP_ = –.1, *p* = .251; gender: Ŷ_gender-SLP_ = –.1, *p* = .509; fluid reasoning: Ŷ_FR-SLP_ = .14, *p* = .175; working memory: Ŷ_WM-SLP_ = .14, *p* = .13; spacing: Ŷ_SP-SLP_ = –.03, *p* = .475). Similarly, spacing (Ŷ_SP-LOG_ = –.03, *p* = .477) did not predict the logarithmic latent variable above and beyond the inclusion of the other predictors. Moreover, the results suggested that older participants had lower plateaus, as indicated by the logarithmic latent variable, compared to younger participants with higher plateaus: Ŷ_age-LOG_ = –.24, *p* = .004. Also, participants with higher fluid reasoning capabilities demonstrated higher plateaus than participants with lower fluid reasoning capabilities: Ŷ_FR-LOG_ = .21, *p* = .032. However, gender (Ŷ_gender-LOG_ = .02, *p* = .906) and working memory (Ŷ_WM-LOG_ = .05, *p* = .550) and spacing (Ŷ_SP-LOG_ = –.03, *p* = .477) did not predict the logarithmic latent variable (see Figure 5a).

The second conditional model with consistency, age, gender, fluid reasoning, and working memory predictors also demonstrated adequate fit indices, as well as significant relationships between the intercept and the log, and the slope and the log variables. Similarly, there was no significant relationship between the intercept and slope variables.

Consistency was not a statistically detectable predictor of baseline performance (Ŷ_CN-INT_ = .07, *p* = .394). However, the results showed that age, fluid reasoning capability, and working memory were statistically significant predictors of the intercept factor. Specifically, older participants had lower baseline scores than younger participants (Ŷ_age-INT_ = –.35, *p* < .001), participants with higher matrix reasoning accuracy scores had higher baseline training capability compared to those with lower scores (Ŷ_FR-INT_ = .25, *p* = .001), similarly, participants with higher working memory capability had higher initial training performance (Ŷ_WM-INT_ = .32, *p* < .001), and lastly individuals that identified as females scored lower at baseline than non-females (Ŷ_gender-INT_ = –.29, *p* = .033). Similar to the model with spacing, the analysis did not detect any statistically significant predictors on the linear slope of the training data (age: Ŷ_age-SLP_ = –.1, *p* = .226; gender: Ŷ_gender-SLP_ = –.1, *p* = .510; fluid reasoning: Ŷ_FR-SLP_ = .14, *p* = .173; working memory: Ŷ_WM-SLP_ = .14, *p* = .131; consistency: Ŷ_CN-SLP_ = –.05, *p* = .408). Moreover, spacing (Ŷ_SP-LOG_ = –.05, *p* = .438) did not predict the logarithmic latent variable above and beyond predictors. Yet, the results suggested that older participants had lower plateaus compared to younger participants with higher plateaus: Ŷ_age-LOG_ = –.25, *p* = .004. Also, fluid reasoning capability demonstrated was positively related to plateaus: Ŷ_FR-LOG_ = .21, *p* = .032. However, gender (Ŷ_gender-LOG_ = .02, *p* = .905) and working memory (Ŷ_WM-LOG_ = .05, *p* = .553) did not predict the logarithmic latent variable (see Figure 5b). Given that, spacing and consistency did not predict performance trajectories on the training paradigm, the interaction between these measures of compliance and individual difference factors in observed behavioral measures was not conducted.

## Discussion

As the field of cognitive training research continues to progress and evolve, there has been a growing interest in understanding the role of individual-level factors (Jaeggi et al., 2014) as well as characterizing mechanisms underlying cognitive training interventions (Green et al., 2019). The current study leveraged an online cognitive training program to examine the predictive validity of individual difference factors in commitment, persistence, and compliance with a cognitive training paradigm. The findings suggest negligible effects of individual difference factors predicting commitment to training, as measured by whether participants advanced beyond sign-up to completing cognitive training sessions. Similarly, there were negligible and inconsistent effects of individual difference factors predicting persistence with cognitive training, as measured by the proportion of training sessions completed. Lastly, compliance factors, such as spacing and consistency with the cognitive training schedule, did not predict engagement with the cognitive training task above and beyond individual difference factors entered as covariates. Taken together, these results add to the complexity of research aimed at identifying individual difference factors accounting for adherence to intervention; albeit, the current study tested these research questions across a large, diverse, and representative sample, which included adults across a broad age range.

Our findings indicate that while some individual difference factors have a statistically detectable impact on predicting commitment to and persistence with training, their effect is very small and inconsistent. As such, the role of these individual difference factors in these subcomponents of adherence remains unclear. Specifically, our findings align with Double and Birney (2016), who also found a negligible relationship between age and adherence, with older adults more likely to persist with cognitive training. However, their study only examined attrition, that is, discontinuation with treatment; while our study looked at persistence, as measured by the number of training sessions completed. It’s worth noting that Turunen et al. (2019) failed to find a significant relationship between age and adherence in their sample of older adults, but they reported that their results approached significance. Overall, it’s possible that older adults are more motivated to complete interventions due to greater availability and fewer commitments to other duties; however, future research will be well served to further explore this effect for replication.

Similarly, we note marginal effects of personality traits on commitment to cognitive training. While we did find an effect of grit and conscientiousness between groups that did and did not advance to training, the effects were negligible and may have been the result of the large sample size and multiple preliminary t-test comparisons. Moreover, the Bonferroni corrected confidence intervals demonstrating a large range in actual effect size and a lower limit close to or equal to 0, suggesting that the effect may be due to chance. We also did not find an effect of grit nor conscientiousness when controlling for other predictors in the planned logistic and multinomial regressions. As such, we do not have strong evidence to suggest that participants that advanced beyond the sign-up stage of the study differed on conscientiousness. Additionally, when controlling for other variables of interest, we report extraversion and agreeableness are inversely related to commitment to training, while emotional stability was directly related to their commitment to training. While statistically significant, the confidence intervals of these effect size of these personality measures straddled equal likelihood (i.e., an odds ratio of 1), which reduced the validity that an effect existed. Double and Birney (2016) also found marginal effects of personality on adherence; however, they report a negative relationship between openness and adherence, which was not replicated in the current study. Moreover, their study found no effects of extraversion, emotional stability, and agreeableness, which could also be a result of differences in how the adherence outcome variable was defined.

Our study also reports no meaningful or consistent relationship between self-reported brain health and commitment to and persistence with cognitive training. More specifically, participants’ self-reported cognitive failures did not identify whether participants were more or less likely to commit to cognitive training; however, there was a statistically tenable but very small effect on persistence with cognitive training. Once again, the effect size here suggested that the individual difference factor presented equal likelihood between naturally occurring groups of completed sessions. Similarly, these results did not appear when focusing on those that completed most of the training sessions and as such the effect of cognitive failures was largely inconsistent. These findings are in line with the findings of Turunen et al. (2019). However, our results differ from those of Cruz et al. (2014), who reported a link between neurodegenerative diseases or brain injuries and adherence. This discrepancy may be due to the small sample size and wide age range in Cruz et al.’s (2014) study. It’s also worth noting that Turunen et al. (2019) focused exclusively on older adults. Additionally, our findings are not in line with those of He et al. (2022), who found that adherence was linked to performance on objective cognitive measures. These differences may be attributed to variations in overall sample size and the diversity within samples recruited across studies.

Previous research has also identified education as a key role in predicting attrition and/or dropout, as well as participants time spent training (Coley et al., 2019; Cruz et al., 2014; Lam et al., 2015). Like the aforementioned individual difference predictors, education level, demonstrated differences in commitment to training as well as persistence with training. More specifically a higher degree was associated with a greater likelihood to commit to cognitive training as well as a greater likelihood of completing most training sessions compared to few training sessions. However, the effects were largely inconsistent when isolating effects between all naturally occurring groups of completed sessions as well as within participants that completed most conditions. Moreover, the effect size for this likelihood of completing most sessions compared to few sessions for individuals with an advanced degree compared to an associate’s degree or below had a significantly large range in the confidence interval. As such, future research would be well served to replicate this finding.

While the present study was the first to examine the relationship between measures of grit (Duckworth & Quinn, 2009), ambition (Duckworth et al., 2007), and socioeconomic status with persistence and commitment to cognitive training, our results did not indicate a significant relationship between these constructs and these components of adherence to cognitive training. Most notably, the study investigated these research questions beyond the extant literature through the liberal inclusion criteria, which resulted in the recruitment of participants throughout adulthood as well as the addition of the aforementioned individual difference factors that had yet to be tested. According to the data, individual difference factors do not seem to play a significant role in adherence, specific to cognitive training, at least not with the measures used in our study.

Overall, we do not find enough evidence to suggest a strong relationship between any of the examined individual differences factors on commitment to and persistence with treatment. It is possible that the small effect sizes of some individual difference predictors were inflated by the sample size combined with numerous predictors; thus, increasing the probability of family-wise error. Similarly, the overall variance explained in models examining individual differences in commitment to training as well as the degree of training sessions completed suggests that these components of adherence may be dependent on factors outside of the characteristics of the participant. Instead, a participant’s experience with the training paradigm may be indicative of some forms of adherence. For instance, research evaluating adherence to behavioral therapy found that participants’ treatment cessation or non-adherence was related to their reported disconnect between capability and needs (Johansson et al., 2015). Similarly, another study examining adherence to an online cognitive behavior therapy identified that optimal levels of motivation were beneficial to treatment adherence (Farrer et al., 2014). Therefore, investing in developing research methodology that investigates approaches to motivating participants, may be worthwhile and fruitful (Deveau et al., 2015; Katz, Jaeggi, et al., 2018; Mohammed et al., 2017; Tullo & Jaeggi, 2022).

There currently exists a plethora of approaches aimed at increasing motivation to participate and adhere to a cognitive training program (Green et al., 2019; Pergher et al., 2020; Tullo & Jaeggi, 2022). For instance, Jaeggi et al. (2014), hypothesized that the degree of the entertainment value of the training could influence differences in participant attrition between treatment and control conditions. To address this, commercialized programs like *Lumosity, Akili Labs*, and *Cogmed* have adopted gamifying cognitive training paradigms (Deveau et al., 2015; Mohammed et al., 2017). Also, *Lumosity* offers participants a variety of different tasks and the ability to choose their preferred training paradigm, while *Cogmed* has utilized personalized coaching to motivate participants (e.g., Nelwan et al., 2018). These efforts to reduce dropouts and attrition and increase adherence are crucial not only for examining individual differences in cognitive training efficacy but also for appropriately evaluating cognitive training mechanisms and approaches. As such, future research in cognitive training will be well served to examine the interaction between individual differences and successful motivational approaches to further tailor cognitive training based on individual needs.

Additionally, the current study did not find any evidence of a relationship between compliance measures and engagement across repeated cognitive training sessions. More specifically, we failed to find a clear relationship between either spacing or consistency and performance trajectories on the training paradigm. The study’s findings are consistent with previous research that also failed to provide any evidence of spacing effects (Jaeggi et al., 2020; Schwaighofer et al., 2015); however, the range of time between tasks did not extend as far as 20 days as in the Wang et al. (2014) study, which is the only cognitive training study that has reported spacing effects. Nevertheless, the current study extends previous research by investigating the effect of spacing as random effects versus previous research’s use of fixed effects. Additionally, and to the best of our knowledge, this is the first study to examine the consistency in cognitive training research as dictated by participants’ autonomy to train at their leisure and availability. While there were no effects of spacing and consistency on learning and engagement with the cognitive training task, it remains unclear whether compliance accounts for the degree of transfer to non-trained tasks.

### Limitations

Although the study provides valuable insights into the predictors of adherence to cognitive training, it is important to acknowledge the limitation in the composition of participants in the naturally occurring groups. Specifically, the number of participants that did not advance past the sign-up stage significantly outmatched the number of participants that did advance to training. The ratio of participants between both groups may have influenced tests examining predictors of these naturally occurring groups. We considered random sampling participants from the larger group of those that signed up but did not advance to training to match the smaller group of participants that did train; however, that procedure would be highly influenced by chance. We also considered propensity-matching participants based on a variable of interest to balance the two groups. However, we encountered difficulty in selecting a variable that would justify the control given that our objective set out to examine the chosen variable’s ability to explain the commitment to training.

Similarly, there is a strong possibility of effects that were not captured here, such as comfort with technology and/or familiarity with brain training games. However, participants of our study voluntarily sought out the research and signed up using their own personal devices. This implies a baseline level of comfort with technology, although we recognize that this assumption may not capture the full spectrum of technology comfort levels. Moreover, previous research has demonstrated associations between adherence measures and individual differences in self-efficacy (Bagwell & West, 2008; He et al., 2022) as well as health literacy (Coley et al., 2019). Despite these limitations, the study highlights the strengths of examining a diverse range of individual difference variables in the context of adherence to cognitive training. The current study has gone above and beyond other similar studies in examining a large range of individual difference factors coupled with a large and diverse sample size to explore said characteristics. As such, future research should continue to explore the role of individual difference factors beyond the factors covered here as well as investigate unknown mediating and/or moderating effects that may explain adherence in cognitive training.

Lastly, while the large sample size coupled with multiple comparisons may have increased the odds of a type 1 error, we have taken considerable efforts and have relaxed the interpretation of the findings to avoid any false positives. Nevertheless, the large sample size and the variety of individual difference factors is a strength of the study examining a research question of this nature. For one, the inclusion of a large sample size in our study has allowed us to ensure the robustness and reliability of our results. We have greater statistical power with a larger sample, enabling us to detect even subtle effects with higher precision. This level of power, in turn, enhances our ability to accurately assess the size of the effects and draw more confident conclusions about the relationships between variables. Second, the incorporation of numerous predictors is a common practice in research studies that aim to explore multiple factors concurrently. By considering a comprehensive set of predictors, we are able to capture the complexity of the phenomenon under investigation and account for a wide range of potential effects. To overcome this obstacle, we have implemented suitable statistical techniques, such as Bonferroni correction, to effectively control for multiple comparisons. These statistical adjustments, which are represented in the confidence intervals, are designed to mitigate the risk of spurious associations, and reduce the likelihood of type I errors and as a result ensures the robustness of our findings, improves our ability to detect even small effects, and provides a comprehensive understanding of the phenomenon under investigation. Taken together, the study design, targeted predictor variables, and statistical corrections affords the opportunity for future research to extrapolate from the models presented here to further characterize the relationship, if any, between individual difference factors and adherence.

## Conclusion

The current study investigates adherence as a key mechanism in cognitive training by characterizing the role of individual difference factors on commitment, persistence, and compliance with a cognitive training regimen. In sum, as individual difference factors assessed here did not reliably predict commitment and persistence with a cognitive training program, there is a need to identify factors that can affect program participation. Moreover, compliance with the cognitive training program, as measured by the average time between cognitive training sessions as well as the average deviance in time between training sessions did not predict engagement with the training paradigm either. Taken together, these results add to the equivocality of findings in research linking individual difference factors to measures of treatment adherence. Nevertheless, there is also a need to motivate participants to complete the cognitive training regimen to improve accuracy in evaluations of the program’s efficacy and effectiveness, which in turn will help reconcile the inconsistency in findings across the field (Green et al., 2019; Katz, Shah, et al., 2018). As such, research in cognitive training should allocate resources and attention to developing methods to systematically motivate participants.

## Data Accessibility Statement

Data corresponding to the analyses are available at the following database: https://osf.io/vw5xj/.

## Additional File

The additional file for this article can be found as follows:

10.5334/joc.315.s1 Supplementary Materials. Supplemental Tables 1 and 2.
