# Supplementary material for: Investigating the Role of Individual Differences in Adherence to Cognitive Training
Source: J Cogn. 2023 Aug 22;6(1):48. doi: 10.5334/joc.315 (PMC10453960; doi:10.5334/joc.315)
Supplement: Supplementary Materials. — Supplemental Tables 1 and 2. [file joc-6-1-315-s1.pdf]

Supplementary Material

Supplemental Table 1

Fit indices for latent growth curve models

**Model**	**L-h**	**AIC**	**BIC**	χ^2	**df**	χ^2**/df**	***p***	**RMSEA [90%CI]**	**CFI**	**TLI**	**SRMR**
Intercept	-5003.24	10050.48	10129.06	3823.51	208	18.38	.000	.257 [.252, .262]	.332	.389	2.02
Linear	-2602.23	5254.46	5343.76	1121.44	205	5.47	.000	.130 [.125, .136]	.831	.843	.211
Logarithmic	-2196.28	4450.57	4554.16	652.97	201	3.24	.000	.092 [.087, .098]	.916	.921	.06
Cond. Log: Spacing	-2048.65	4185.31	4339	817.28	286	2.86	.000	.087 [.082, .09]	.916	.915	.051
Cond. Log: Consistency	-2048.15	4184.3	4338	817.11	286	2.85	.000	.087 [.082, .093]	.916	.915	.051

Note. Logliklihood, AIC, BIC, chi-square test of fit with corresponding p-values, and fit indices are presented per model.

Supplemental Table 2

Intercepts (means), variances and covariances of latent variables in latent growth curve models

	Intercept		Slope		Logarithmic	
**Model**	**Mean (SE), *p***	**SD^2 (SE), *p***	**Mean (SE), *p***	**SD^2 (SE), *p***	**Mean (SE), *p***	**SD^2 (SE), *p***
Unconditional Logarithmic	2.25 (0.02), $p < .001$	0.06 (0.01), $p < .001$	0.04 (0.01), $p < .001$	0.01 (0.00), $p < .001$	0.15 (0.16), $p < .001$	0.05 (0.01), $p < .001$
Conditional Logarithmic: Spacing	2.24 (0.11), $p = .327$	0.03 (0.01), $p < .001$	0.04 (0.05), $p = .327$	0.01 (0.00), $p < .001$	0.68 (0.42), $p = .105$	0.52 (0.1), $p < .001$
Conditional Logarithmic: Spacing	2.43 (0.11), $p < .001$	0.03 (0.01), $p < .001$	0.05 (0.05), $p = .320$	0.01 (0.00), $p < .001$	0.69 (0.43), $p = .105$	10.52 (0.1), $p < .001$

Note. Estimates, standard errors, and p-values for means and variances of latent variables (i.e., intercept, slope, and logarithmic asymptote) are presented across unconditional logarithmic and conditional logarithmic (i.e., spacing and consistency) models.

Covariances of latent variables in latent growth curve models

	Intercept - Slope	Intercept - Logarithmic	Slope - Logarithmic
**Model**	**Estimate (SE), *p***	**Estimate (SE), *p***	**Estimate (SE), *p***
Unconditional Logarithmic	0.00 (0.00), $p = .608$	0.03, (0.01), $p < .001$	-0.01 (0.00), $p = .006$
Conditional Logarithmic: Spacing	0.00 (0.00), $p = .132$	0.06, (0.02), $p = .002$	-0.03 (0.01), $p = .001$
Conditional Logarithmic: Spacing	0.00 (0.00), $p = .133$	0.06, (0.02), $p = .002$	-0.03 (0.01), $p = .001$

Note. Estimates, standard errors, and p-values for the covariance between latent across unconditional logarithmic and conditional logarithmic (i.e., spacing and consistency) models.
